# Deoxyelephantopin and Isodeoxyelephantopin as Potential Anticancer Agents with Effects on Multiple Signaling Pathways

**DOI:** 10.3390/molecules22061013

**Published:** 2017-06-21

**Authors:** Tahir Mehmood, Amara Maryam, Hamed A. Ghramh, Muhammad Khan, Tonghui Ma

**Affiliations:** 1 College of Basic Medical Sciences, Dalian Medical University, Dalian 116044, China; tahir_nanotech@yahoo.com (T.M.); amara_khan2015@yahoo.com (A.M.); 2 Research Center for Advanced Materials Science, King Khalid University, Abha 61413, P.O. Box 9004, Saudi Arabia; hamedsa@hotmail.com; 3 Department of Biology, Faculty of Science, King Khalid University, Abha 61413, P.O. Box 9004, Saudi Arabia

**Keywords:** deoxyelephantopin, isodeoxyelephantopin, *Elephantopus scaber*, *Elephantopus carolinianus*, apoptosis, cancer, inflammation

## Abstract

Cancer is the 2nd leading cause of death worldwide. The development of drugs to target only one specific signaling pathway has limited therapeutic success. Developing chemotherapeutics to target multiple signaling pathways has emerged as a new prototype for cancer treatment. Deoxyelephantopin (DET) and isodeoxyelephantopin (IDET) are sesquiterpene lactone components of “*Elephantopus scaber and Elephantopus carolinianus*”, traditional Chinese medicinal herbs that have long been used as folk medicines to treat liver diseases, diabetes, diuresis, bronchitis, fever, diarrhea, dysentery, cancer, and inflammation. Recently, the anticancer activity of DET and IDET has been widely investigated. Here, our aim is to review the current status of DET and IDET, and discuss their anticancer activity with specific emphasis on molecular targets and mechanisms used by these compounds to trigger apoptosis pathways which may help to further design and conduct research to develop them as lead therapeutic drugs for cancer treatments. The literature has shown that DET and IDET induce apoptosis through multiple signaling pathways which are deregulated in cancer cells and suggested that by targeting multiple pathways simultaneously, these compounds could selectively kill cancer cells. This review suggests that DET and IDET hold promising anticancer activity but additional studies and clinical trials are needed to validate and understand their therapeutic effect to develop them into potent therapeutics for the treatment of cancer.

## 1. Introduction

Cancer is the second leading cause of mortality and remains a major threat to public health worldwide [1]. According to the World Health Organization (WHO) statistical estimation, 27 million new cancer cases and 17.5 million cancer deaths are likely to occur by 2050 [2]. Only in Europe and the United States, the frequency of cancer incidence is 2.6 million cases per year [3]. Cancer is characterized by unchecked and uncontrolled proliferation of cells caused by the dysfunction of various essential genes which code for vital proteins including anti-apoptotic proteins, transcription factors, tumor suppressers, growth factors, and growth factor receptors [3,4]. Cancer is developed due to multiple aberrations rather than a single abnormality. Therefore, treating cancer by using mono-target chemical agents is not an effective approach. However, cancer is a preventable ailment and the concept of chemoprevention by natural products, especially of plant origin, is gaining more importance as it is a safe and cost effective alternative approach for cancer treatment [1]. Cancer treatment and chemoprevention by phyto-compounds has shown promising results as phyto-compounds target multiple mechanisms which are central to cancer development and proliferation.

Throughout human history, natural products, particularly plant-based systems, have been extensively used in the treatment of wide spectrum ailments. Plants are considered as the basic milestone to lay the foundation of traditional medicine systems. The medicinal use of 1000 plant-derived formulations which are still in use for the treatment of parasitic infections and inflammations has been documented, dating back to around 2600 BC, in Mesopotamia. Dating back to 1500 BC, 700 formulations of plant origin have been documented in “Ebers Papyrus”. Likewise, the Chinese Materia Medica, Indian Ayurvedic system, Greeks, and Romans have contributed a lot of essential medicinal formulations to the development of the use of plant-derived products against various diseases in the ancient eras [5,6]. Natural products are the richest source of outstanding chemical and structural diversity and represent the most fruitful authenticated approach of small molecule drug discovery. The importance of natural products can be valued by the fact that 87% of all categorized human disease, including cancer, bacterial/parasitic infections, and inflammations are being treated with natural product formulations [3]. According to the WHO report, 80% of the global population still relies on plant-derived medicines for their primary health care [1,3,6,7]. Statistical reports show that more than 60% of commercially available anticancer drugs are derived from natural sources including plants, marine organisms, and micro-organisms [8,9]. Moreover, about 20% of cancer incidence and 200,000 cancer-related deaths can be prohibited with more consumption of fruits and vegetables [1,10,11]. As cancer is poorly defined in folklore and traditional medicines, despite skepticism, plants have long been used to treat cancers in traditional medicines [5,6]. More than 3000 plant species have been used to treat cancers [12].

There are several reasons that have put forward plants as an important source of potentially active anticancer compounds in the field of drug discovery and development. First, the complexity, molecular and structural diversity of natural compounds make plants an ideal candidate for drug discovery and development, as nature has been applying evolutionary pressure for 3 billion years to develop and screen the unique molecular and structural diversity in plants [13,14]. Second, the plant-derived anticancer compounds target multiple signaling cascades which are central to cancer progression [4]. Third, plants have not been fully explored for discovering potential anticancer compounds. It is estimated that about 2.5 to 5 million species of plants exist on earth and only about 6% and 15% of the 300,000 identified species of higher plants have been systematically investigated for their pharmacological and phyto-chemical values, respectively [5,6]. Fourth, the production of bioactive anticancer compounds by plants often exceeds the current capacity of synthetic chemistry and can meet the demands of clinical application [13]. Beside the above mentioned properties, another reason that makes plants an ideal source of potential anticancer compounds is the absorption affinity of phyto-compounds. Phyto-compounds are absorbed quickly as compared to that of synthetic drugs [15]. Plant-derived natural compounds are already in clinical trials for treating cancers with progressively growing demand. Despite all these advantages, some pharmaceutical companies have decreased the use of natural products due to the development of new approaches to drug discovery such as combinatorial chemistry and computer-based molecular modeling design and also concerns about intellectual rights and difficulties in access and the supply of sources [4]. However, none of them can be a substitute for natural products in drug discovery and development. The aim of this review is to summarize and discuss the current status, natural sources, biological activities, molecular targets, anticancer activity, mechanism of action, and usefulness of the natural bioactive compounds “Deoxyelephantopin (DET) and isodeoxyelephantopin (IDET)” in anticancer drug development. DET and IDET, sesquiterpene lactone compounds, are active components of *Elephantopus scaber and Elephantopus carolinianus*. DET and IDET reveal anticancer activity against several human cancer cell lines by targeting multiple signaling pathways which are central to cancer development and propagation. 

## 2. Natural Sources and Biological Activities

The sesquiterpene lactone compounds, DET and IDET, are major components of *Elephantopus scaber and Elephantopus carolinianus* which belong to the family Asteraceae [16,17,18]. *Elephantopus scaber* Linn, a medicinal plant of the Asteraceae family, is commonly known as “elephant’s food” in English, “tutup bumi” in Malaysian, “didancao” in Chinese, and “gobhi” in Hindi [19,20,21]. The herb is widely distributed all over the world especially in East Asia, Southeast Asia, Africa, Australia, India, and South America [20,21,22]. It showed various biological activities including anti-bacterial, anti-diabetic, anti-inflammatory, wound healing, hepatoprotective, and anticancer activity [23,24,25]. For years, the root syrup of *Elephantopus scaber* has been used to treat liver injuries and hepatitis in Brazil. In China, traditional medicinal syrup “Yi-GanYin” has been commonly consumed by people to protect the liver from cirrhosis, fatty accumulation, hemangioma, cancer, and hepatitis B, whereas in Taiwan, the herbal medicinal formulation “Teng-Khia-U” has been used by people to treat edema, nephritis, chest pain, scabies, and pneumonia [26,27,28,29,30]. These formulations are made up of a few medicinal herbs containing *Elephantopus scaber* as an important constituent. 

**Figure 1:**
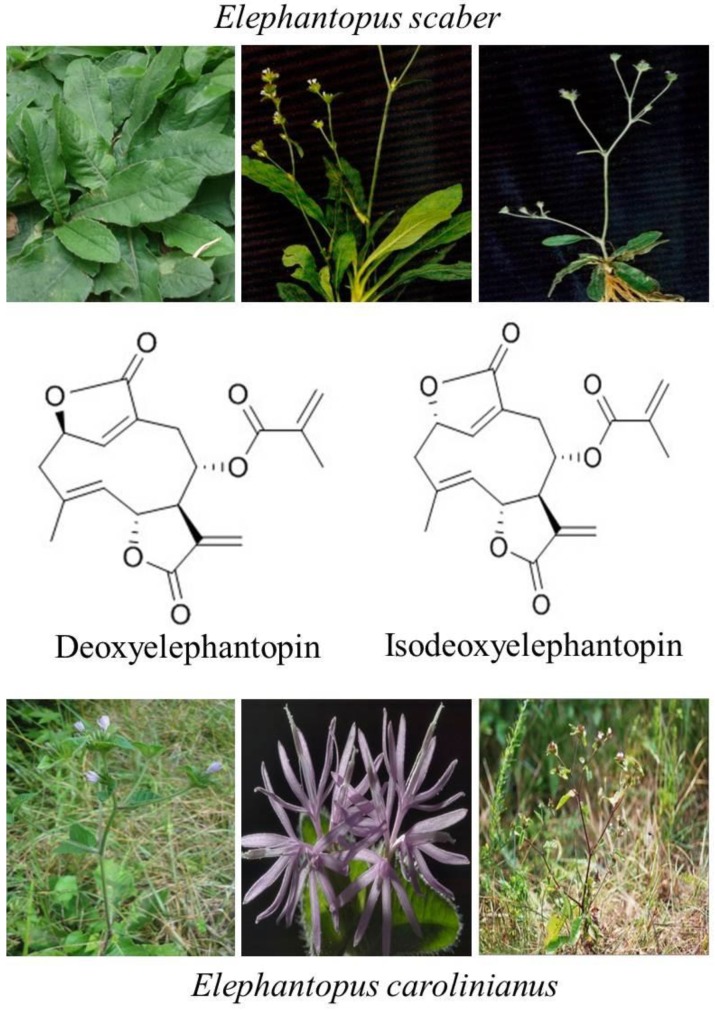
Chemical structure and natural sources of deoxyelephantopin (DET) and isodeoxyelephantopin (IDET).

The sesquiterpene lactones are regarded as chemotaxonomic markers of the genus Elephantopus and their biological activities are mainly attributed to sesquiterpene lactones [20]. DET and IDET are major sesquiterpene lactone components extracted from *Elephantopus scaber* and *Elephantopus carolinianus* [17,31]. Recent studies have shown that they are active compounds with multiple pharmacological and antitumor activities. DET has shown the antitumor activity against nasopharyngeal [32,33], cervical [24,34], breast [22,24,35], lung [22,24], colon [24,36,37], and liver cancer [38], whereas its isomer IDET has potentiated leukemia, breast cancer and lung cancer [39] by targeting multiple pathways. Interestingly, DET has shown relatively less cytotoxic activity against CCD841CoN normal colon cells [36] and lymphocytes at concentrations which are toxic to A549 lung cancer cells [40]. Similarly, IDET has shown specific cytotoxic activity toward T47D breast cancer cells and A549 cells and not toward normal lymphocytes [22]. The chemical structures and natural sources of DET and IDET are shown in Figure 1.

## 3. Targeting Cancer Cells by Apoptosis Pathways

The term “apoptosis” was first used in 1972 by Kerr JFR et al. with the discovery of a new pattern of cell death. Since then apoptosis has been extensively studied and valued as a mechanism of regulated cell death. It is labeled synonymously with the term “programmed cell death” as it is regulated by several interlinked cell signaling cascades to death [41]. Morphologically, characteristic hallmarks of apoptotic cell death include cell shrinkage, chromatin condensation (pyknosis), nuclear fragmentation (karyorrhexis), and apoptotic bodies formation containing deceased cell’s cytoplasmic and nuclear material [24,42]. The malfunctioning of apoptosis regulators may lead to tumorigenesis. Several molecular and biochemical pathways are deregulated in cancer cells to halt apoptosis. Thus, the activation of the apoptosis regulatory machinery by novel therapeutic compounds containing the potential to induce apoptosis in cancer cells may provide an opportunity to develop drugs for cancer treatment. 

Extensive studies have revealed that anticancer natural compounds can induce apoptotic-mediated cell death in various cancers [43,44]. DET and IDET, sesquiterpene lactone compounds, have been shown to induce apoptosis in various cancer cells through multiple mechanisms, including mitochondrial dysfunction, reactive oxygen species (ROS) Induction, Bcl-2 family protein modulation, mitotic arrest induction, inhibition of nuclear factor kappa B (NF-κB), and signal transducers and activators of transcription 3 (STAT3) activation [22,24,34,35,36,37,38,39]. 

### 3.1. Targeting Cancer Cells by Cell Cycle Arrest-Mediated Apoptosis

Cell cycle arrest, an increase in the proportion of cells at a particular checkpoint of the cell cycle, is one of the major causes of cell death in cancer cells. Each phase of the cell cycle is regulated by the corresponding interactions of various cyclins with their respective cyclin-dependent kinases (CDKs) which ensure that each phase of the cell cycle is accomplished properly before proceeding to the next phase [45]. Cyclin-dependent kinase inhibitors (CDKIs) such as p21 negatively regulate CDKs and play an active role in the regulation of cell cycle arrest at particular checkpoints [46,47,48]. Several studies have evidenced that clinical chemotherapeutic drugs such as taxol and vinca alkaloids cause cell cycle arrest at specific checkpoints and induce apoptosis [45].

**Figure 2:**
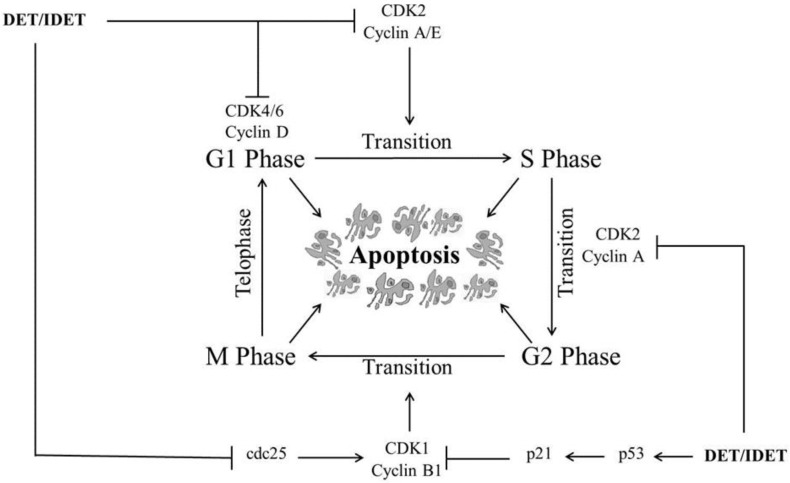
A schematic representation of DET- and IDET-induced cell cycle arrest at multiple phases in different cancer cell lines. DET and IDET inhibit S phase and/or G2/M phase transition by down-regulating the expression of cyclin-dependent kinase 1 (CDK1), CDK2, CDK4, CDK6, cyclin A2, cyclin D1, cyclin D3, cyclin E2, cyclin B1, and cdc2 while up-regulating the expression of p21 (CDK inhibitor) and p53, a tumor suppresser gene which regulates cell cycle arrest at different phases by inducing p21;├ Inhibition, ↑ Up-regulation.

DET and IDET have been shown to induce cell cycle arrest at S phase and/or G2/M phase in various cancers. In HCT116 cells, DET induced cell cycle arrest at S phase preventing the transition from S phase to G2/M phase by down-regulating CDK2, CDK4, cyclin A2, cyclin D1, cyclin E2, and cyclin B1 in a dose dependent manner [36]. In SiHa cells, DET induced G2/M phase arrest by down-regulating cyclin B1 and cdc2 while up-regulating p21 (CDK inhibitor) and p53, a tumor suppresser gene which regulates G2/M phase arrest by inducing p21 and lowering the intracellular level of cyclin B1 [34]. It has been shown that IDET selectively induced G2/M phase arrest with a 2 h drug treatment in the CNE1 and SUNE1 cell lines, while it did not induce cell cycle arrest at any phase in NP69 normal nasopharyngeal cells [32]. DET induced apoptosis in TS/A cells through G2/M phase arrest by deregulating the expressions of cyclin B1 and Phospho-CDK1. DET up-regulated the expression of p21 which controls the cell cycle arrest at G2/M phase [35]. Moreover, DET induced G2/M phase arrest in A549 [40] and HeLa cells [49] in a dose-dependent manner. It has been shown that DET induced cell cycle arrest at S phase dose-dependently and G2/M phase soon after DET treatment, which gradually decline at a high concentration in CNE cells. DET induced G1/S phase arrest by down-regulating CDK4, CDK6, and cyclin D1 and D3 which are the major regulators of G1/S phase arrest while it induced G2/M phase arrest by down-regulating the expression of cdc2, cdc25 dose-dependently, and cyclin B1 at high concentrations in CNE cells, leading cells to apoptosis [33]. Various mechanisms are implicated by DET and IDET to arrest multiple checkpoints of the cell cycle which accelerate apoptosis in different cell lines, as shown in Figure 2.

### 3.2. Targeting Cancer Cells by Extrinsic Apoptosis

Activation of cell surface receptors (death receptors) triggers the extrinsic apoptotic pathway. The death receptors (DR) such as fibroblast-associated antigen (Fas), TNF-related apoptosis-inducing ligand (TRAIL), tumor necrosis factor receptor-1 (TNFR1), DR3, DR4, DR5, and DR6 transmit signals from extracellular ligand binding domains to cytoplasmic death domains [50,51]. Upon ligand binding, a death inducing signaling complex (DISC) is formed by the interaction of DRs with the Fas-associated death domain (FADD) and procaspase-8. Within DISC, procaspase-8 is activated by auto-proteolysis and becomes free which, in turn, either activates the type 1 extrinsic apoptotic pathway by activating downstream effector caspase-3 or the type 2 extrinsic apoptotic pathway by the truncation of Bid, a pro-apoptotic member of the Bcl-2 protein family [51,52,53].

**Figure 3:**
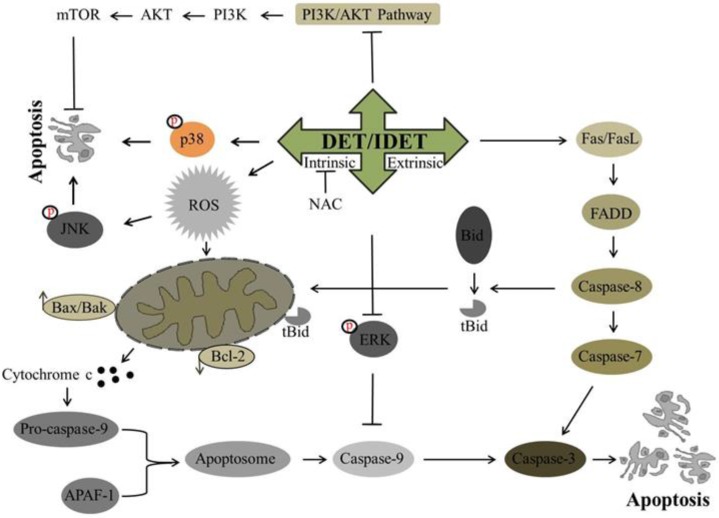
A schematic representation of DET- and IDET-induced apoptosis in different cancer cell lines. DET and IDET trigger the activation of extrinsic apoptosis by activating caspase-8 which in turn either initiates the type 1 extrinsic apoptotic pathway by activating downstream effector caspase-3 or the type 2 extrinsic apoptotic pathway by truncation of Bid. DET and IDET induce intrinsic apoptosis by dissipating mitochondrial membrane potential and modulating the expression of Bcl-2 family proteins which results in the activation of caspase-3. Subsequently, activated caspase-3 leads to apoptosis by substrate cleavage. DET activates c-Jun N-terminal kinase (JNK) and p38 and inhibits the activation of Phosphatidylinositol-3-Kinase (PI3K)/AKT/ mammalian target of rapamycin (mTOR). DET inhibits the activation of extracellular signal-regulated kinase (ERK)-mitogen-activated protein kinase (MAPK) and activates caspase-9 and induces apoptosis; ├ Inhibition, ↑ Up-regulation, ↓ Down-regulation.

Recently, DET has been investigated for its potential anti-cancer activity against many cancer cell lines. It has been shown to trigger extrinsic apoptosis in different cancers including cervical cancer, lung cancer, and nasopharyngeal cancer. In SiHa cells [34] and A549 cells [40], DET has been reported to induce the activation of procaspase-8 while in CNE cells, DET has been shown to trigger type I and type II extrinsic apoptosis by up-regulating the expression of FasL, active caspase-10, -8, -3, and Bid truncation at high concentration. Truncated Bid enters into mitochondria, modulates Bcl-2 family proteins, and causes further release of cytochrome c. Both type I and II extrinsic apoptotic pathways merge at the caspase-3 level, induce PARP cleavage, and ultimately result in apoptosis [33]. In TS/A cells, DET induced extrinsic apoptosis by the activation of caspase-8 through TNF/TNFR-mediated activation, as DET increased the expression level of TNF-R1 protein rather than increasing the expression level of the Fas and FasL proteins [35]. The underlying mechanism of DET and IDET for inducing extrinsic apoptosis is shown in Figure 3.

### 3.3. Targeting Cancer Cells by Mitochondrial-Mediated/Intrinsic Apoptosis

It is well elucidated that mitochondria are an important component of apoptosis regulators and play a vital role in the execution of apoptosis [12]. The intrinsic apoptotic pathway is triggered by various extracellular and intracellular stimuli such as chemotherapeutic agents, DNA damage, hypoxia, depletion of various growth factors, induction of oxidative stress, and irradiation. These stimuli generate signals that have particular targets within cells such as the down regulation of anti-apoptotic Bcl-2 and Bcl-xL proteins and the activation of Bax, Bak, and Bad proteins to form a pore structure in the mitochondrial membrane [54,55]. Bcl-2 family protein modulation results in the loss of mitochondrial membrane potential (ΔΨm) which, in turn, causes the release of pro-apoptotic proteins including cytochrome c and apoptosis inducing factors (AIF) from the inter-membrane space of mitochondria into the cytosol where cytochrome c activates caspase-9 by interacting with apoptotic protease activating factor-1 (Apaf-1). Subsequently, Caspase-3 is activated by activated caspase-9 and is led to cleave cellular proteins such as the inhibitor of caspase-activated DNase (ICAD) and PARP, leading to nucleosomal DNA fragmentation and thus apoptosis [4,54].

The apoptotic effect of DET and IDET has been investigated in different cancer cell lines. Different studies have shown that DET and IDET induce apoptosis in different cancers including cervical cancer, lung cancer [40], breast cancer [56], colorectal cancer, nasopharyngeal cancer, and liver cancer by modulating the Bcl-2 family proteins. In SiHa cells, DET has been reported to enhance the Bax/Bcl-2 ratio on the mRNA level, release cytochrome c in the cytosol, and activate caspase-9, -7, and -3, and cleave PARP [34]. The anticancer activity of DET [33] and IDET [32] has been reported in CNE and CNE1 cells, respectively, with increased expression of pro-apoptotic Bax, Bad, Bok, Bmf, and PUMA, decreased expression of anti-apoptotic Bcl-2 and Bcl-xL protein, and increased expression of activated caspase-12, -9, -7, -3, and PARP cleavage. DET induces apoptosis by targeting caspase-9, -3, and PARP cleavage in TS/A cells [35]. It has been shown that DET also induces intrinsic apoptosis in HCT116 [36], HepG2 cells [38] and HeLa cells [49] by modulating pro-apoptotic protein Bax and anti-apoptotic protein Bcl-2, and by activating caspase-9 and -3 and the cleavage of PARP. Accumulative data demonstrate that DET and IDET hold the potential to induce intrinsic apoptosis by modulating the Bcl-2 family proteins. Based on targeting the Bcl-2 family proteins, DET and IDET can be explored further in various cancer cell lines to develop them as effective chemotherapeutics. The underlying mechanism of DET and IDET to induce intrinsic apoptosis has been shown in Figure 3.

### 3.4. Targeting Cancer Cells by ROS-Mediated Apoptosis

Reactive oxygen species (ROS) can be defined as oxygen containing reactive chemical entities [4,37]. It is well established that cancer cells exhibit higher oxidative stress than normal cells which plays a vital role in cell survival, proliferation, metastasis, angiogenesis, and the disturbance of cell death signaling and drug resistance [4,57]. Under normal physiological conditions, reactive oxygen species produced inside the cells are neutralized by the anti-oxidant system of the cells which maintains the cells at a stable redox balance. Over production of ROS destabilizes the oxidative balance and causes oxidative damage to the cellular components including DNA, lipids, and proteins, which ultimately leads to apoptosis [58]. Accumulative evidence have proven that despite tumor endorsing, the ROS producing biochemical property of cancer cells could be exploited by phytochemicals through additional ROS generation above the toxic threshold level to kill cancer cells selectively [4,38].

DET has been reported to induce apoptosis in cervical carcinoma (SiHa), hepatocellular carcinoma (HepG2), and murine mammary adenocarcinoma (TS/A), while IDET has been reported to induce apoptosis in nasopharyngeal carcinoma (CNE1) by targeting ROS metabolism through various mechanisms. In HepG2 cells, DET has been shown to induce ROS dependent apoptosis. DET induced ROS generation, reduced the activity of thioredoxin reductase (TrxR), depleted the intracellular level of glutathione (GSH), disrupted the mitochondrial membrane potential, enhanced DNA fragmentation, and decreased the translocation of NF-κB into the nucleus. N-Acetyl-L-Cysteine (NAC) pretreatment which is a ROS scavenger reversed the effect of DET in HepG2 cells indicating the involvement of ROS in DET-induced apoptosis [38]. In TS/A cells, DET induced apoptosis via ROS generation and c-Jun N-terminal kinase (JNK) activation. It has been shown that oxidative stress and JNK activation was required for DET-induced cell death as the reversal of DET-mediated expression of c-Jun, p21, and the activation of JNK and p21 was seen with NAC pretreatment [35], as shown in Figure 3. In CNE1 cells, the effect of IDET was found to be associated with ROS generation, mitochondrial membrane dysfunction, DNA fragmentation, G2/M phase arrest, and up-regulation of anti-tumor inflammation factors such as cytokines (IL-12a) and interferon (INF-α, INF-β). The reversal of all of these IDET-induced events was observed with the supplementation of NAC except for INF-α and INF-β, indicating that IDET targeted multiple pathways with or without inducing ROS generation [32].

## 4. Targeting Cancer Cells by Regulating Multiple Signaling Pathways and Transcription Factors

As cancer is a multi-step process which is developed due to multiple aberrations rather than a single abnormality, treating cancer by using mono-target chemical agents is not an effective approach. It may be a better therapeutic approach to target multiple signaling pathways that may lessen the hazards of drug resistance, which is a major drawback of anticancer drugs intended to block a specific signaling pathway. DET and IDET have been explored for their inhibitory effects on multi-target signaling pathways in different cell lines. 

### 4.1. Mitogen-Activated Protein Kinases (MAPKs) Pathway

Mitogen-activated protein kinases (MAPKs), ubiquitous serine/threonine protein kinases family with other members including extracellular signal-regulated kinase-1 and 2 (ERK1/2), c-Jun N-terminal kinase (JNK)/stress activated protein kinase (SAPK), and p38, play a vital role in the regulation of several process such as cell proliferation, differentiation, and apoptosis. The activation of ERK1/2 has been implicated with tumor progression, drug resistance, and poor prognoses with a shorter overall survival rate [59,60]. It has been shown in multiple studies that in various human cancers, this pathway is deregulated, and it is considered as a prime target for anticancer drug development. In SiHa cells, DET has been reported to suppress the activation of ERK1/2 by inhibiting phosphorylation. It is shown that ERK-MAPK phosphorylates caspase-9 at the Thr125 residue resulting in the inhibition of caspase-9 activation, DET increased caspase-9 activity by inhibiting the activation of ERK1/2 which showed that the apoptotic effect of DET was induced by inhibiting the ERK pathway [34]. The effect of DET on the ERK pathway might be cell type specific, as it increased the expression of ERK1/2 in CNE cells [33]. JNKs and p38 MAPKs play an essential role in the signaling cascade events which orchestrate cellular responses to various types of cellular stress [60]. DET activated SAPK-JNK and p38 via phosphorylation, resulting in the modulation of Bcl-2 family proteins and decreasing the Bcl2/Bax ratio in SiHa cells [34]. In CNE cells, DET dose-dependently increased the expression of phosphorylated-JNK (p-JNK) indicating the involvement of the JNK pathway in apoptotic cell death. 

It has been reported by in vivo studies that DET showed a hepatoprotective effect by targeting the JNK pathway. In mice, LPS/D-galactosamine (D-GalN)-induced liver damage resulted in activation of JNK1/2. The increased phosphorylation of JNK1/2 induced by LPS/D-GalN was significantly decreased by DET treatment. In TNF-α-mediated hepatocyte apoptosis, JNK1 degrades FADD-like IL-1beta-converting enzyme (FLICE)/caspase-8-inhibitory protein (FLIP) and JNK2 activates capase-8. It has been shown that in mice injected with LPS/D-GalN, hepatocytes underwent TNF-α-mediated apoptosis by caspase-8-dependent activation of Bid and JNK-induced activation of Bim. DET suppressed the activation of JNK, caspase-8, Bid, caspase-3, and PARP, and reversed LPS/D-GalN-induced liver damage in mice [61]. Accumulative data suggest that DET not only up-regulates JNK in cancer cells to induce apoptosis but also targets the up-regulated JNK in liver injuries caused by different stimuli. This dual property makes DET not only a commendable chemopreventive but also an effective therapeutic agent to treat cancers. The mechanisms implicated by DET to target MAP kinases are shown in Figure 3.

### 4.2. PI3K/AKT/mTOR Pathway

Phosphatidylinositol-3-Kinase (PI3K)/AKT belongs to the serine/threonine kinase protein family and plays an important role in tumor growth, enhancing cell survival by stimulating cell proliferation and inhibiting apoptosis. Activation of AKT by phosphorylation at threonine 308 (Thr 308) and serine 473 (ser473) residues activates several transcription factors and increases the transcription of anti-apoptotic and pro-survival genes. AKT also activates Mammalian target of rapamycin (mTOR) protein kinase, a serine/threonine kinase by phosphorylation [62,63,64]. mTOR is a key regulator of cell growth, proliferation, and apoptosis, and its over expression leads to poor prognosis. The inhibition of AKT activation is considered as a potential stratagem in cancer cells. DET decreases the phosphorylation of AKT and mTOR in SiHa cells [34], whereas its effect on AKT might be cell type specific because in CNE cells DET increased the expression of the phosphorylated form of AKT [33]. The mechanism implicated by DET to target the PI3K/AKT/mTOR pathway is shown in Figure 3.

### 4.3. STAT3 Signaling Pathway

Signal transducers and activators of transcription 3 (STAT3), a member of the STAT protein family, is a cytoplasmic transcription factor and is aberrantly activated in several cancers such as lymphoma, breast, lung, ovarian, prostate, and liver cancers [65]. Phosphorylation of STAT3 at tyrosin-705 or serine 727 triggers the activation of STAT3 which can be mediated by growth factor receptors, cytokines such as interleukin 6 (IL-6), cytoplasmic kinases such as Janus activated kinases, and Src family Kinases and ABL family kinases [65,66]. Upon activation, two phosphorylated STAT3 form a dimer and are translocated into the nucleus, where they control numerous essential genes and act as a chief regulator of cell proliferation, differentiation, metastasis, angiogenesis, apoptosis, and immune response [67,68].

**Figure 4:**
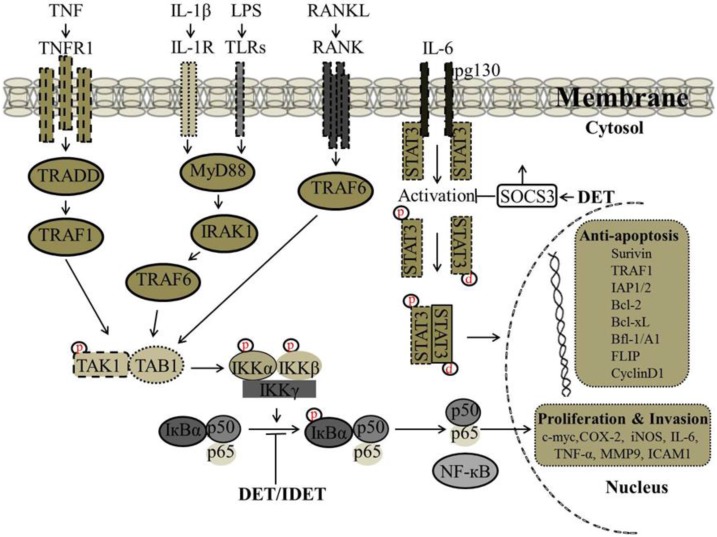
Proposed mechanism by which DET and IDET inhibit nuclear factor kappa B (NF-κB) and signal transducers and activators of transcription 3 (STAT3) activation and NF-κB and STAT3-regulated gene expression involved in cell proliferation and invasion. DET and IDET inhibited the activation of NF-κB induced by various inflammatory stimuli such as (tumor necrosis factor (TNF), lipopolysaccharide (LPS), interleukin-1 beta (IL-1β) which activates NF-κB through different pathways. DET and IDET inhibited NF-κB activation at the same step of all these stimuli. Inhibition of NF-κB activation was associated with decreased phosphorylation and the degradation of IκB-α and upstream activation of IκB kinase-alpha/-beta (IKK-α/-β) and IKK kinase activities. DET and IDET down-regulated the expression of NF-κB-regulated gene products involved in invasion such as matrix metalloproteinase (MMP-9) and intercellular adhesion molecule 1 (ICAM-1); cell proliferation such as cyclooxygenase-2 (COX-2), cyclin D1, and cellular-Myc (c-Myc); and anti-apoptosis such as inhibitor of apoptosis protein-1/-2 (IAP-1/-2), B-cell lymphoma 2 (Bcl-2), B-cell lymphoma-extra-large (Bcl-xL), Bcl-2-related protein A1 also known as Bfl-1/A1, TNF receptor-associated factor (TRAF1), FADD-like IL-1beta-converting enzyme (FLICE)/caspase-8-inhibitory protein (FLIP), and survivin). DET inhibits the activation of STAT3 by reducing phosphorylation at tyrosine-705 and up-regulates the suppressor of cytokine signaling 3 (SOCS3) which is a major component for the negative regulation of the IL-6 signaling cascade; ├ Inhibition, ↑ Up-regulation.

It has been reported that there is a correlation between wild type p53 expression and STAT3 phosphorylation at tyrosine-705, as p53 could inhibit the constitutive activation of pSTAT3 (tyrosine-705). In SiHa cells, DET up-regulated the expression of p53 and inhibited the phosphorylation of p-STAT3 at tyrosine-705 and down-regulated the expression of STAT3-regulated gene products including Bcl-2 and Bcl-xL [34]. Accumulative evidence suggests that lipopolysaccharides (LPS) induce STAT3 phosphorylation leading to the production of pro-inflammatory cytokines including IL-6. Activation of STAT3 by IL-6/IL-6R is critical to inflammation. In male ICR mice liver insulted with LPS/D-GalN, the decreased protein level of suppressor of cytokine signaling 3 (SOCS3), a major component for negative regulation of the IL-6 signaling cascade, was significantly increased with DET treatment which resulted in suppression of STAT3 phosphorylation at tyrosine-705 and decreased the pSTAT3/STAT3 ratio significantly, suggesting that DET can protect against acute liver inflammation through the IL-6/STAT3 pathway [61]. The proposed mechanism by which DET inhibits STAT3 activation and STAT3-regulated gene expression involved in cell proliferation and invasion are shown in Figure 4.

### 4.4. NF-κB Signaling Pathway

The NF-κB pathway plays an important role in transactivation of several important genes involved in tumor cell proliferation, invasion, metastasis, apoptosis, and angiogenesis [69]. The NF-κB family of transcription factors is composed of five members such as NF-κB1 (p105/p50), NF-κB2 (p100/p52), RelA (p65), RelB, and c-Rel [67,70]. Under normal physiological conditions, NF-κB is retained in the cytoplasm by association with its endogenous inhibitors of κBs (IκBs). The activation of NF-κB can be triggered by various stimuli including tumor-necrosis factor (TNF), interleukin 1 (IL-1), interleukin 6 (IL-6), endotoxins, carcinogens, tumor promoters, microbial pathogens, free radicals, lipopolysaccharide (LPS), and radiation (UV-light, X-rays, γ-rays) [3]. The activation of NF-κB is a multistep process that is usually initiated with the phosphorylation of inhibitors of κBs (IκBs) by IκB kinases (IKK) which is composed of two catalytic subunits IKK-α and IKK-β and one regulatory subunit IKK-γ, resulting in ubiquitination or proteasomal degradation of IκBs [3,71]. Once IκB-α is phosphorylated, the p50/p65 complex, the most common active form of the NF-κB complex, is formed and transported into the nucleus where it initiates transcription of specific oncogenes and anti-apoptotic genes (survivin, Bcl-2 family protein, IAP) [70]. 

It has been shown that DET and IDET inhibited constitutive activation of NF-κB in human multiple myeloma (MM.1S and U266) and head and neck squamous cell carcinoma (SCC4 and LICR-LON-HN5) cells, which are known to constitutively express active NF-κB. In the MCF-7, H1299, and HL60 cell lines, DET and IDET inhibited activation of NF-κB induced by various inflammatory stimuli (TNF, LPS, IL-1β, and Phorbol 12-myristate 13-acetate (PMA)) which activate NF-κB through different pathways indicating that DET and IDET inhibited NF-κB activation at the same step of all of these stimuli. The inhibition of NF-κB activation was associated with decreased phosphorylation and degradation of IκB-α and the upstream activation of IKK-α/-β and IKK kinase activities. DET and IDET down-regulated the expression of NF-κB-regulated gene products involved in invasion (MMP-9 and ICAM-1), cell proliferation (COX-2, cyclin D1, and c-Myc), and anti-apoptosis (IAP1, IAP2, Bcl-2, Bcl-xL, Bfl-1/A1, TRAF1, FLIP, and survivin) [39]. Recently, we have shown the effect of DET on NF-κB activity in HepG2 liver cancer cells. DET inhibited constitutive and inducible (TNF-α- and Gemcitabine-induced) activation of NF-κB in HepG2 cells in a dose-dependent manner. The inhibition of both constitutive and induced-activation of NF-κB by DET was found to be associated with decreased phosphorylation of IκB-α [38]. In TS/A cells, DET has been shown to suppress TNF-α-induced NF-κB activity and down-regulate NF-κB-regulated gene products involved in invasion such as MMP-2 and MMP-9, consequently suppressing the migration and invasion of TS/A cells in vitro and in vivo. DET-mediated blockage of NF-κB-p65 was associated with the formation of hydrogen bonds between the carbonyl group at position 16 of DET and the Lys122 residue of NF-κB-p65 [35]. The proposed mechanism by which DET and IDET inhibit NF-κB activation and NF-κB-regulated gene expression involved in cell proliferation and invasion are shown in Figure 4.

## 5. Conclusions and Future Perspectives

In this review, we have summarized the recent progress of DET and IDET in various cancer cell lines. Collective data from different studies indicate that DET and IDET, sesquiterpene lactone compounds, show potent anticancer activity and the following observations make these two structural isomers effective therapeutic agents for the treatment of various cancers: (I) DET and IDET show a broad-spectrum of anticancer activity toward various human cancers; (II) they have been shown to induce cell death by activating apoptotic machinery in cancer cells; (III) they inhibit multiple signaling pathways such as NF-κB, STAT3, ERK, and PI3k/AKT/mTOR which are constitutively activated in human cancers and play major roles in cancer progression and drug resistance; (IV) Unlike pharmacological drugs which are severely toxic to healthy body cells, DET and IDET, as the major components of *Elephantopus scaber* and *Elephantopus carolinianus* which have long been used in traditional Chinese medicines to cure various ailments, are considered safe chemotherapeutic compounds for the treatment of cancer. *Elephantopus scaber* and *Elephantopus carolinianus* are widely distributed all over the world especially in East Asia, Southeast Asia, Africa, Australia, India, and South America. The aforementioned findings illustrate that DET and IDET may become potential lead compounds to treat cancers, however, preclinical and clinical trials are still required to explore the entire spectrum of anticancer activity of DET and IDET to endorse their further utility as potent anticancer agents.
